# Hippocampal Excitatory Synaptic Transmission and Plasticity Are Differentially Altered during Postnatal Development by Loss of the X-Linked Intellectual Disability Protein Oligophrenin-1

**DOI:** 10.3390/cells11091545

**Published:** 2022-05-05

**Authors:** Noemie Cresto, Nicolas Lebrun, Florent Dumont, Franck Letourneur, Pierre Billuart, Nathalie Rouach

**Affiliations:** 1 Neuroglial Interactions in Cerebral Physiology and Pathologies, Center for Interdisciplinary Research in Biology, Collège de France, CNRS UMR 7241, INSERM U1050, Labex Memolife, Université PSL, 75005 Paris, France; noemie.cresto@college-de-france.fr; 2 Institut de Psychiatrie et de Neurosciences de Paris, INSERM U1266, Université de Paris Cité, 75014 Paris, France; nicolas.lebrun@inserm.fr; 3 UMS IPSIT, Université Paris-Saclay, 92296 Châtenay-Malabry, France; florent.dumont@universite-paris-saclay.fr; 4 Institut Cochin, INSERM U1016, CNRS UMR8104, Université de Paris Cité, 75014 Paris, France; franck.letourneur@inserm.fr

**Keywords:** intellectual disability, Oligophrenin-1, synaptic transmission, plasticity, hippocampus, development

## Abstract

Oligophrenin-1 (OPHN1) is a Rho-GTPase-activating protein (RhoGAP), whose mutations are associated with X-linked intellectual disability (XLID). OPHN1 is enriched at the synapse in both pre- and postsynaptic compartments, where it regulates the RhoA/ROCK/MLC2 signaling pathway, playing a critical role in cytoskeleton remodeling and vesicle recycling. *Ophn1* knockout (KO) adult mice display some behavioral deficits in multiple tasks, reminiscent of some symptoms in the human pathology. We also previously reported a reduction in dendritic spine density in the adult hippocampus of KO mice. Yet the nature of the deficits occurring in these mice during postnatal development remains elusive. Here, we show that juvenile KO mice present normal basal synaptic transmission, but altered synaptic plasticity, with a selective impairment in long-term depression, but no change in long-term potentiation. This contrasts with the functional deficits that these mice display at the adult stage, as we found that both basal synaptic transmission and long-term potentiation are reduced at later stages, due to presynaptic alterations. In addition, the number of excitatory synapses in adult is increased, suggesting some unsuccessful compensation. Altogether, these results suggest that OPHN1 function at synapses is differentially affected during maturation of the brain, which provides some therapeutic opportunities for early intervention.

## 1. Introduction

Intellectual disability (ID) is a neurodevelopmental disorder starting before the age of 18 leading to alterations in intellectual functioning (IQ < 70) and adaptative behavior, including social, conceptual, and practical abilities [1]. The pathology affects 1–2% of the worldwide population, is often misdiagnosed, and induces high costs for the healthcare system [2], pointing to the need to develop new therapeutical strategies for this neurological disease. The causes of ID are complex and include environmental and genetic origins.

The second most frequent form of genetic ID in males, after Down syndrome, results from mutations in chromosome X-linked genes [3]. A large fraction of ID genes code for proteins involved in synapse function, and it appears that common features of ID and, more specifically, X-linked ID (XLID) are dysfunctions of gene expression (chromatinopathy) or the synapse (synaptopathy) [4,5,6]. The Oligophrenin-1 gene (*Ophn1*) is involved in a syndromic form of X-linked ID (XLID) with cerebellar hypoplasia, and missense mutations in *Ophn1* are also associated with schizophrenia and autism spectrum disorders [7,8,9], making this gene a strong candidate (category 2) according to the Simons Foundation Autism Research Initiative (SFARI) score [10]. OPHN1 is enriched at the synapse and regulates the RhoA/ROCK/MLC2 signaling pathway through its RhoGAP domain, playing a critical role in cytoskeleton remodeling [7]. *Ophn1* knockout (KO) mice are today considered as a mouse model for ID, as they present a human-like syndromic form of XLID apart from the cerebellar phenotype. These mice display an enlargement of brain ventricles and defects in working and spatial memory, as well as social interactions and hyperactivity [11]. In neurons, OPHN1 regulates neurogenesis, dendritic spine maturation, and axon guidance [12,13,14,15]. At the presynaptic site, OPHN1 loss of function impairs synaptic vesicles (SV) endocytosis, recycling, and pool replenishment [16]. In *Ophn1* KO mice, the number of SVs available for release is reduced in the hippocampus [17], and an increase in short-term synaptic depression is observed at CA1 Schaffer collateral synapses [14]. At the postsynaptic level, OPHN1 loss of function impairs AMPA-R subunit internalization. Consequently, NMDA-dependent long-term depression (LTD) is altered at CA1 Schaffer collateral synapses from *Ophn1* KO mice [16]. Most of these studies focused on neuronal alterations at the adult stage. However, since we observed a developmental decrease in dendritic spines in these animals [18], we here studied during postnatal development the nature of the synaptic alterations in both juvenile and adult *Ophn1* KO mice. We report a differential pattern of functional alterations, pointing to a developmental aggravation of the phenotype resulting in impaired basal synaptic transmission and long-term potentiation (LTP) in adult *Ophn1* KO mice.

## 2. Materials and Methods

### 2.1. Animals

All procedures on animals were performed according to the guidelines of European Community Council Directives of 01/01/2013 (2010/63/EU) and French regulations (Code Rural R214/87-130). Experimental procedures were approved by our local ethics committee (CEEA No. 059, Paris Centre et Sud) and registered with the French Research Ministry (APAFIS No. 2017112817353516). Because the *Ophn1* gene is located on the X chromosome, only males *Ophn1* KO mice (*Ophn1*^−/y^) (KO: B6.129P-Ophn1^tm1Bill^) and their wildtype (WT) littermates (*Ophn1*^+/y^) were included in the study [11]. All applicable international, national, and/or institutional guidelines for the care and use of animals were followed. Adult mice were housed under a 12 h light/12 h dark circle, with ad libitum access to food and water. All efforts were made to minimize the number of used animals and their suffering, taking into consideration the “3Rs recommendation” (replacement, reduction, and refinement) for animal experimentation.

### 2.2. Ex Vivo Electrophysiology

#### 2.2.1. Tissue Preparation

Mice were sacrificed by cervical dislocation and decapitation. The hippocampi were rapidly isolated and sectioned at 4 °C using a vibratome (model VT1200S, Leica, Bannockburn, IL, USA) in an artificial cerebrospinal fluid (aCSF). For experiments on juvenile mice (25 to 30 days old), acute transverse hippocampal slices (400 μm) were prepared as previously described [19]. Slices were maintained at room temperature in a storage chamber in an aCSF containing (in mM) 119 NaCl, 2.5 KCl, 2.5 CaCl_2_, 1.3 MgSO_4_, 1 NaH_2_PO_4_, 26.2 NaHCO_3_, and 11 glucose, saturated with 95% O_2_ and 5% CO_2_, for at least 1 h prior to recording. For experiments on adult mice (3 to 4 months old), hippocampi were cut in a cold solution containing (in mM) 250 d-sucrose, 25 sodium bicarbonate (NaHCO_3_), 3 potassium chloride (KCl), 1 calcium chloride (CaCl_2_), 10 magnesium chloride (MgCl_2_), and 10 d-glucose, oxygenated with carbogen (95% oxygen (O_2_) and 5% carbon dioxide (CO_2_)). Slices were maintained at room temperature in a storage chamber for 30 min in the same solution and then transferred in normal aCSF as previously described. A proportion of slices were used for electrophysiological experiments, while the others were fixed in 2% paraformaldehyde (2% PFA diluted in PBS, Waltham, MA, USA) for histological experiments.

#### 2.2.2. Extracellular Field Recordings

**Figure 1:**
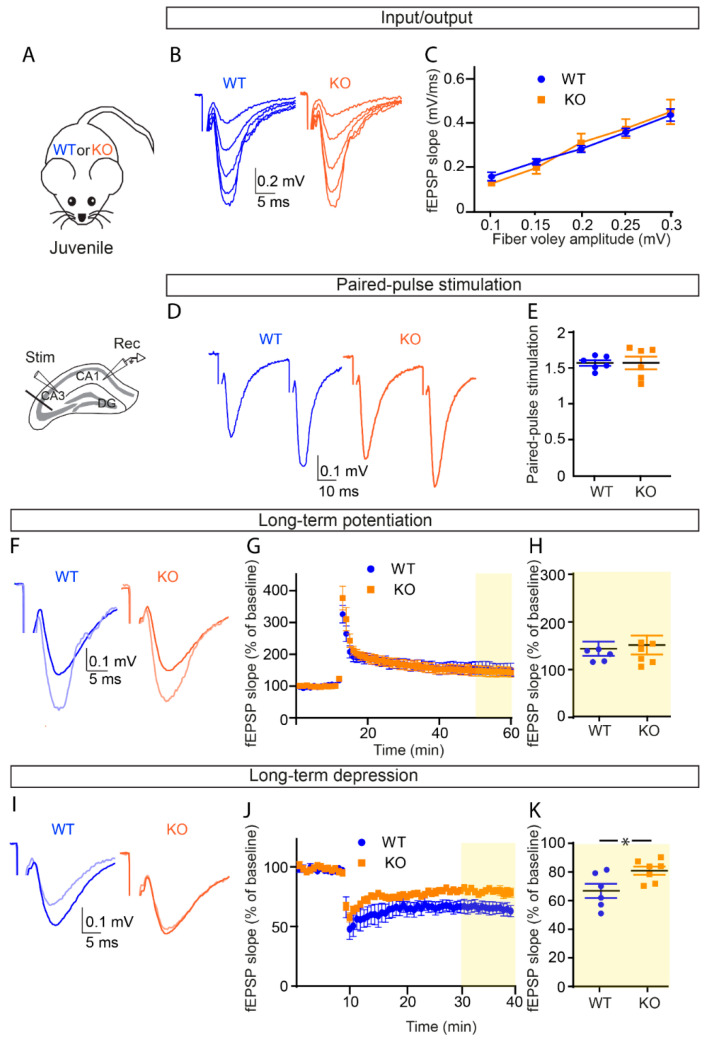
Selective impairment in long-term depression with no change in basal excitatory synaptic transmission and long-term potentiation in juvenile OPHN1-deficient mice. (**A**) Scheme of the hippocampus illustrating the arrangement of the stimulating electrode, to activate the Schaffer collaterals, and of the neuronal recording electrode (extracellular), to record fEPSPs evoked by the stimulation in the CA1 area of *Ophn1* KO mice (KO) juvenile mice or WT littermate. (**B**,**C**) The input/output curve shows that basal excitatory synaptic transmission is unchanged in KO mice (*n* = 5 slices from five mice) as compared to WT mice (*n* = 4 slices from four mice) (two-way ANOVA, genotype: *p* = 0.9990; F(1,7) = 1.58 × 10^−6^). (**D**,**E**) Paired-pulse facilitation (PPF) is unchanged in KO mice (*n* = 5 slices from five mice) as compared to WT mice (*n* = 6 slices from four mice) (unpaired *t*-test, *p* = 0.9893; DF = 10). (**F**–**H**) Long-term potentiation (LTP) was measured by field potential recordings (fEPSPs) at CA1 Schaffer collateral synapses in 1 month old KO mice as compared to WT littermates. Averaged fEPSPs after the stimulation train were normalized to baseline values before high-frequency stimulation. The yellow box highlights the last 10 min of the recording (50–60 min). (**H**) The histogram represents the mean normalized fEPSP slope measured during the last 10 min of the recording, as indicated by the yellow box in (**G**). LTP was unaffected in KO mice (*n* = 7 slices from six mice) compared to WT mice (*n* = 6 slices from four mice). (**G**) Two-way ANOVA, genotype: *p* = 0.9827; F(1,11) = 0.0004926; (**H**) Mann–Whitney test, *p* = 0.8357; U = 19). Sample traces represent averaged field potentials before (dark blue and dark orange) and 50–60 min after tetanization (light blue and light orange). (**I**–**K**) LTD was measured by field potential recordings (fEPSPs) at CA1 Schaffer collateral synapses in 1 month old KO mice as compared to WT littermates. Averaged fEPSPs after the stimulation were normalized to baseline values before stimulation. The yellow box highlights the last 10 min of the recording (30–40 min). (**K**) The histogram represents the mean normalized fEPSP slope measured during the last 10 min of the recording, as indicated by the yellow box in (**J**). LTD was reduced in KO mice (*n* = 7 slices from six mice) compared to WT mice (*n* = 6 slices from four mice). (**J**) Two-way ANOVA, genotype: *p* = 0.0481; F(1,11) = 4.942; (**K**) unpaired *t*-test, *p* = 0.0278; DF = 11). Asterisks indicate statistical significance (* *p* < 0.05). Sample traces represent averaged field potentials before (dark blue and dark orange) and 30–40 min after the LTD induction (light blue and light orange).

**Figure 2:**
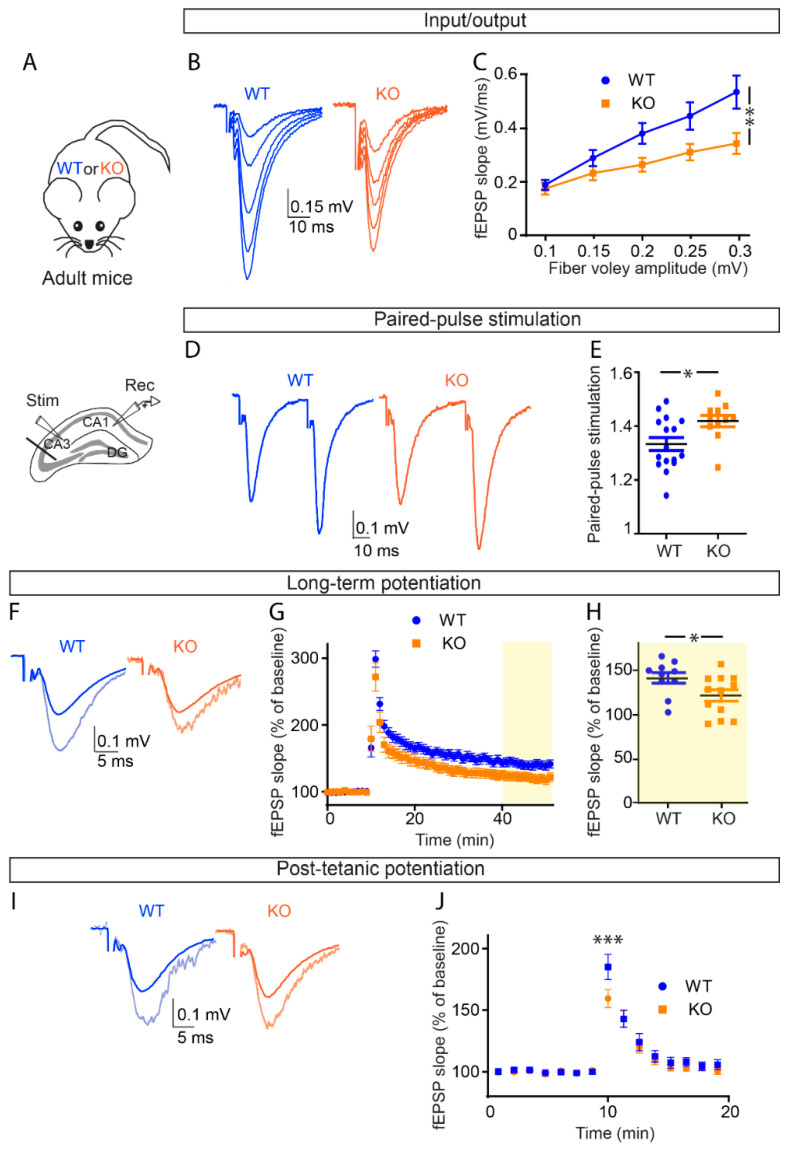
Basal excitatory synaptic transmission is reduced in adult OPHN1-deficient mice. (**A**) Scheme of the hippocampus illustrating the arrangement of the stimulating electrode, to activate the Schaffer collaterals, and of the neuronal recording electrode (extracellular), to record fEPSPs evoked by the stimulation in the CA1 area of KO mice or WT littermates. (**B**,**C**) The input/output curve shows that basal excitatory synaptic transmission is reduced KO mice (*n* = 7 slices from five mice) as compared to WT littermates (*n* = 7 slices from five mice) (two-way ANOVA, interaction (genotype × fiber volley amplitude, *p* < 0.0001; Bonferroni post hoc test at 0.3 mV, *p* = 0.0022). (**D**,**E**) Paired-pulse facilitation (PPF) is increased in KO adult mice (*n* = 11 slices from five mice) as compared to WT littermates (*n* = 16 slices from five mice), suggesting a reduction in the release probability (unpaired *t*-test, *p* = 0.0194; DF = 25). (**F**–**H**) Long-term potentiation (LTP) was measured by field potential recordings (fEPSPs) at CA1 Schaffer collateral synapses in KO mice as compared to WT littermates. (**G**,**H**) Average fEPSPs at 35 min after these trains were normalized to baseline values before high-frequency stimulation. Reduced LTP was observed in KO mice (*n* = 12 slices from five mice) compared to WT littermates (*n* = 10 slices from five mice). (**G**,**H**) Two-way ANOVA, genotype: *p* < 0.0001; F(1,1040) = 131.8; unpaired *t*-test, *p* = 0.0407; DF = 20. (**I**,**J**) Post-tetanic potentiation (PTP) was measured by field potential recordings (fEPSPs) at CA1 Schaffer collateral synapses in KO mice as compared to WT littermates. (**J**) Average fEPSPs after stimulation train were normalized to baseline values before high-frequency stimulation. Reduced PTP was observed in KO mice (*n* = 11 slices from five mice) compared to WT littermates (*n* = 13 slices from five mice, Two way ANOVA, interaction (genotype × time: *p* = 0.0212; F(14,304) = 1.952; Bonferroni post hoc test, *p* = 0.0002). Sample traces represent averaged field potentials before and 15–20 min after tetanization (light blue and light orange). Asterisks indicate statistical significance (* *p* < 0.05; ** *p* < 0.01; *** *p* < 0.0001).

For field recordings, slices were transferred to a submerged recording chamber mounted on an Olympus BX51WI microscope equipped for infrared differential interference (IR-DIC) microscopy and perfused with aCSF containing picrotoxin (100 µM) at a rate of 1.5 mL/min by a peristaltic pump at 30–32 °C. A cut was made between the CA3 and CA1 regions to prevent the propagation of epileptiform activity. Glass pipettes, pulled with a horizontal puller (Sutter Instrument, Novato, CA, USA) and filled with aCSF, were used to stimulate CA1 Schaffer collaterals and were placed at a distance of ~200 µm from the area where evoked field excitatory postsynaptic potentials (fEPSP) were recorded (Figures 1 and 2). fEPSPs were recorded and filtered (low pass at 1 kHz) with an Axopatch 200 A amplifier (Axon Instruments, Union City, CA, USA), digitized at 10 kHz with an A/D converter (Digidata 1322 A, Axon Instruments), and stored and analyzed on a computer using Pclamp9 and Clampfit9 software (Molecular Devices, San Jose, CA, USA). Baseline evoked responses were monitored online over 10 min, and only slices with stable fEPSP amplitudes were included. Input–output (I–O) relations for fEPSPs were measured at the start of each experiment by applying a series of stimuli of increasing intensity to the Schaffer collaterals. Paired-pulse facilitation (PPF) of fEPSPs was evoked by delivery of two stimuli at an interval of 40 ms, and it was measured by dividing the peak amplitude of the second response to the one of the first response. Long-term potentiation (LTP) was induced by tetanic stimulation of Schaffer collaterals (two trains of 100 Hz for 1 s, 20 s apart). Long-term depression (LTD) was induced by 1 Hz stimulation of Schaffer collaterals over 15 min. Post-tetanic potentiation was induced by tetanic stimulation of the Schaffer collaterals (two trains of 100 Hz for 1 s, 20 s apart) in the presence of 10 µM CPP ((*RS*)-3-(2-Carboxypiperazin-4-yl-)propyl-1-phosphonic acid).

### 2.3. Histology, Image Acquisition, and Analysis

#### 2.3.1. Immunohistochemistry

Slices (400 µm) were blocked for 1 h at room temperature with phosphate-buffered saline (PBS, Sigma-Aldrich, Saint-Louis, MO, USA) containing 1% Triton X100 (Sigma-Aldrich, Saint-Louis, MO, USA) and 0.2% gelatin (Sigma-Aldrich, Saint-Louis, MO, USA), incubated 48 h at 4 °C with primary antibodies in the blocking solution, washed three times with PBS, incubated overnight with secondary antibodies in the blocking solution, and then washed three times with PBS before mounting with Abberior mounting medium (Abberior Instruments GmbH, Göttingen, Germany). To label excitatory synapses, we used the presynaptic marker VGlut1 (in magenta, 1/200 Synaptic Systems, Göttingen, Germany, #135511) and the postsynaptic marker Homer-1 (in yellow, 1/200, Synaptic Systems, Göttingen, Germany, #160003). To stain inhibitory synapses, we used the presynaptic marker Gad67 (in cyan, 1/200, Millipore, Burlington, MA, USA, #ABN904) and the postsynaptic marker Gephyrin (in red, 1/200, Synaptic Systems, Göttingen, Germany, #147011). Appropriate secondary antibodies were then used (goat anti-mouse star red (1/200, Abberior, Göttingen, Germany, #1001-500UG), goat anti-rabbit 594 (1/200, Invitrogen, Waltham, MA, USA, #A32740), goat anti-rabbit star red (1/200, Abberior, #1002-500UG), and goat anti-mouse 594 (1/200, Invitrogen, #A32742)).

#### 2.3.2. Image Acquisition

For the analysis of the number of synapses, images were taken using a super-resolution custom upright stimulated emission depletion (STED) microscope (Abberior Instruments GmbH, Göttingen, Germany). The microscope is based on a Scientifica microscope body (Slice Scope, Scientifica, Uckfield, UK) equipped with an Olympus 100X/1.4NA ULSAPO objective lens. It comprises a scanner design featuring four mirrors (Quad Scanner, Abberior Instruments GmbH, Göttingen, Germany), with 488 nm, 561 nm, and 640 nm excitation lasers available (Abberior Instruments, pulsed at 40/80 MHz). A laser at 775 nm (MPB-C, pulsed at 40/80 MHz) is used to generate STED beams. The conventional laser excitation and STED laser beams are superimposed using a beam-splitter (HC BS R785 lambda/10 PV flat, AHF Analysetechnik, Tübingen, Germany). Common excitation power with pulsed excitation ranges from 10–20 µW with STED power intensities of up to 200 mW in the focal plane. Nine regions of interest of about 400 to 700 µm^3^ were imaged in CA1.

#### 2.3.3. Images Analysis

Confocal images were then deconvolved using Huygens software and combined with the STED images in one file for analysis. The analysis was performed in ImageJ with an in-house developed plugin. In brief, maxima intensities were identified in the STED images and then compared to the deconvolved confocal images to remove false-positive punctae. When two punctae from pre- (VGlut1 or Gad67) and postsynaptic markers (Homer-1 or Gephyrin) were within 300 µm of each other, a synapse was assigned as a pixel in between the two punctae.

### 2.4. Transcriptomic Data

Total RNA from adult (9 weeks old) mouse hippocampi (*n* = 12 for each genotype) was reverse-transcribed, labeled, and probed on three Agilent microarray 8 × 60K slices, as previously described [20].

Principal component analysis (PCA) revealed two outliers in controls that were excluded from the subsequent supervised ANOVA, considering three variable parameters (genotype, time, and microarray slices) [21]. Raw and normalized data are available at https://www.ncbi.nlm.nih.gov/geo/query/acc.cgi?acc=GSE190891, accessed on 24 December 2021). 

#### Gene Set Expression Analysis

Normalized data were processed for gene set expression analyses according to a procedure [22] using lists of genes involved in the regulation of glutamatergic synaptic transmission or in neurotransmission and genes encoding post and pre-synaptic proteins described in molecular signature database v7.5.1 updated 1 January 2022 (http://www.gsea-msigdb.org/gsea/msigdb/index.jsp).

### 2.5. Statistical Analysis

All data are expressed as the mean ± *SEM*. Prior to statistical comparison, normality tests and variance analysis (Shapiro–Wilk test) were performed, and the appropriate two-sided parametric or nonparametric statistical test was used. Statistical significance for within-group comparisons was determined by two-way analysis of variance (ANOVA) followed by Bonferroni’s post hoc test, whereas Mann–Whitney or unpaired *t*-tests were used for between-group comparisons (DF, U, and *p* are reported in the figure legends). For ANOVA, the genotype effect and the F factor are reported in the figure legends (F (DFn, DFd)). Statistical analysis was performed using Prism 9.3.1 (Graphpad Software, CA, USA).

## 3. Results

### 3.1. OPHN1 Deficiency Does Not Alter Basal Excitatory Synaptic Transmission in the Hippocampus of Juvenile Mice

To examine the effect of OPHN1 deficiency on synaptic strength in juvenile mice, we evaluated basal evoked synaptic transmission at CA1 Schaffer collateral synapses in acute hippocampal slices from 3- to 4-week-old *Ophn1* KO mice (KO) (Figure 1A). By comparing the amplitude of the presynaptic fiber volley (input) to the slope of the excitatory field potential (fEPSP) (output), we found that juvenile KO mice had normal excitatory synaptic transmission compared to WT littermates (Figure 1B,C, two-way ANOVA). We further investigated the presence of presynaptic alterations in these mice by assessing paired-pulse facilitation (PPF), a form of short-term plasticity sensitive to changes in presynaptic release probability. Consistent with the intact basal synaptic transmission, we found normal PPF in juvenile mice (Figure 1D,E, unpaired *t*-test), indicating no change in the probability of presynaptic release.

### 3.2. OPHN1 Deficiency Differentially Alters Long-Term Synaptic Plasticity in Juvenile Mice

We next investigated whether juvenile mice display alterations in long-term synaptic plasticity. To do so, we assessed both long-term potentiation (LTP) and depression (LTD) using extracellular field potential recordings. We found that LTP, induced by brief tetanic stimulation (two 1 s trains of 100 Hz, 20 s apart) of Schaffer collaterals, was unchanged in KO mice compared to their WT littermates (Figure 1F–H; two-way ANOVA, genotype: *p* = 0.9827, Figure 1G; Mann–Whitney test, *p* = 0.8357, WT, *n* = 6 slices from four mice; KO, *n* = 7 slices from six mice, Figure 1H), displaying proper induction and maintenance. In contrast, the magnitude of LTD, induced by 1 Hz stimulation of Schaffer collaterals (1 Hz over 15 min) was decreased in KO juvenile mice compared to their WT littermates (Figure 1I–K; two-way ANOVA, genotype: *p* = 0.481, Figure 1K; unpaired *t*-test, *p* = 0.0278, WT, *n* = 6 slices from four mice; KO, *n* = 7 slices from six mice, Figure 1K). Altogether, these data indicate that OPHN1 deficiency leads to differential alteration of long-term synaptic plasticity in juvenile mice, with a selective impairment in LTD.

### 3.3. OPHN1 Deficiency Impairs Hippocampal Excitatory Synaptic Transmission and Long-Term Potentiation in Adult Mice

We then investigated whether the electrophysiological alterations induced by OPHN1 deficiency in juvenile mice worsens in adult mice. In contrast to our results obtained in juvenile mice, adult mice deficient for OPHN1 displayed impaired basal excitatory transmission at CA1 Schaffer collateral synapses, as assessed by input–output curves using field potential recordings (Figure 2A–C). We indeed found a ~37% reduction in excitatory synaptic transmission for fiber volley amplitude of 0.3 mV in KO mice compared to 3 to 4 month old WT littermates (fEPSP slope: WT: 0.54 ± 0.06 mV/ms (*n* = 7 slices from five mice) vs. KO: 0.34 ± 0.04 mV/ms (*n* = 7 slices from six mice), two-way ANOVA and Bonferroni’s multiple comparisons test, *p* = 0.0022, Figure 2B,C). To investigate whether the defect in synaptic transmission in OPHN1-deficient adult mice resulted from presynaptic alterations, we assessed transmitter release probability by recording PPF of field potentials. We found PPF to be increased in KO adult mice compared to WT littermates (WT: 1.33 ± 0.02, *n* = 16 slices from five mice, vs. KO: 1.42 ± 0.02, *n* = 11 slices from five mice; *p* = 0.0194, unpaired *t*-test, Figure 2D,E), indicating a decrease in the probability of presynaptic release.

Because OPHN1 deficiency reduces efficacy of CA1 Schaffer collateral synapses in adult mice, we next investigated its involvement in long-term synaptic plasticity. We found that the magnitude of LTP, induced by brief tetanic stimulation of Schaffer collaterals, was reduced by ~15% in KO adult mice (WT: 141.6 ± 6.15%, *n* = 10 slices from five mice; vs. KO: 121.7 ± 6.52%, *n* = 12 slices from five mice; *p* = 0.0407, unpaired *t*-test, Figure 2F–H). We then tested whether this impairment resulted from a defect in LTP induction due to presynaptic alterations. To do so, we assessed post-tetanic potentiation (PTP), which is a transient potentiation of synaptic transmission resulting from massive synaptic glutamate release induced by the tetanic stimulation and recorded while blocking NMDA receptors (CPP, 10 µM) to prevent LTP induction. Using recordings of field potentials, we found that OPHN1-deficient mice displayed reduced magnitude of PTP (normalized fEPSP slope: WT: 185% ± 10.18%, *n* = 13 slices from five mice vs. KO: 159.5 ± 8.13%, *n* = 11 slices from five mice, two-way ANOVA, Bonferroni’s multiple comparison, *p* = 0.0002, Figure 2I,J). Taken together, the impairments in basal synaptic transmission and LTP found in adult, but not juvenile KO mice indicate that the alteration in synaptic strength resulting from presynaptic defects emerges during late postnatal development.

### 3.4. OPHN1 Deficiency Selectively Increases the Number of Excitatory Synapses in Adult Mice

**Figure 3:**
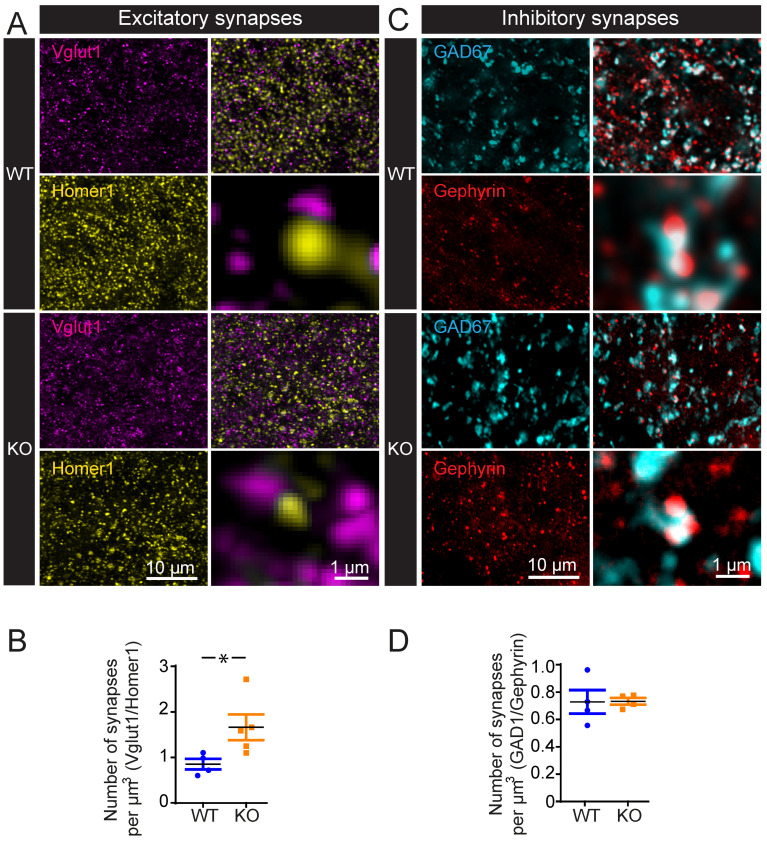
OPHN1 deficiency induces a selective increase in the number of excitatory synapses in adult mice. (**A**,**C**) Representative images of excitatory synapses, labeled with Vglut1 (magenta) and Homer1 (yellow), and inhibitory synapses, labeled with Gad677 (cyan) and Gephyrin (red). (**B**) Quantification of the numbers of excitatory synapses (WT: *n* = 17,674 synapses from *n* = 4 mice, KO: *n* = 29,377 synapses from *n* = 5 mice) (unpaired *t*-test, *p* = 0.0469; DF = 7). (**D**) Quantification of the number of inhibitory synapses (WT: *n* = 5125 synapses from *n* = 4 mice, KO: *n* = 5155 synapses from *n* = 4 mice) (unpaired *t*-test, *p* = 0.9633; DF = 6). Asterisks indicate statistical significance (* *p* < 0.05).

Adult mice deficient for OPHN1 have been reported to display an alteration in dendritic spines, with a reduction in length and density primarily affecting mature mushroom spines [11]. To evaluate whether this leads to an alteration in the number of synapses, we quantified, using super-resolution STED imaging, the number of excitatory and inhibitory synapses in the hippocampus of adult mice. Synapses were labeled with a combination of pre- and postsynaptic markers, Vglut1 and Homer1, respectively, for excitatory synapses (Figure 3A) and Gad67 and gephyrin, respectively, for inhibitory synapses (Figure 3C). An ImageJ plugin was generated to automatically detect and quantify the number of synapses (see Section 2). We found that the number of excitatory synapses was increased in KO mice as compared to WT littermates (WT: 0.8523 ± 0.1160/µm^3^, *n* = 17,674 from four mice vs. KO: 1.6625 ± 0.3643/µm^3^, *n* = 29,377 from five mice; *p* =0.0469, unpaired *t*-test, Figure 3B).

OPHN1 deficiency has been reported to lead to impairments in the maturation and function of both excitatory and inhibitory synapses [23]. We, thus, investigated whether adult KO mice also display an alteration in the number of inhibitory synapses. We, however, found that the number of inhibitory synapses (Figure 3D) was not altered in KO mice (WT: 0.7314 ± 0.0857/µm^3^, *n* = 5125 from four mice vs. KO: 0.7356 ± 0.0247/µm^3^, *n* = 5155 from four mice; *p* = 0.9633, unpaired *t*-test). These data suggest that the selective increase in the number of CA1 excitatory synapses in OPHN1-deficient mice may be a compensation for the altered excitatory transmission.

### 3.5. Transcriptomic Analysis Reveals That Glutamatergic Synaptic Transmission Is Altered in OPHN1-Deficient Mice

**Figure 4:**
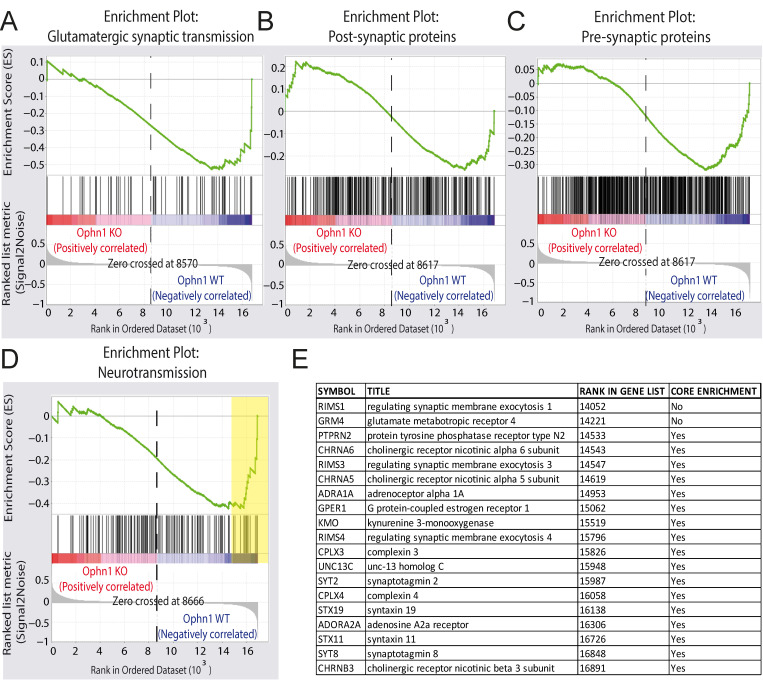
Gene set enrichment analysis reveals that OPHN1 deficiency in adult mice induces a downregulation of genes involved in neurotransmission. Gene set enrichment analysis (GSEA) comparing molecular signatures for (**A**) glutamatergic transmission (69 genes), (**B**) postsynaptic gene membrane (252 genes), (**C**) presynaptic compartment (465 genes), and (**D**) neurotransmission (140 genes) were performed on microarray transcriptomes of hippocampi from KO (*n* = 12) and WT (*n* = 10) mice. (**E**) List of 17 neurotransmission genes that mostly contribute to the genset enrichment in control (normalized enrichment score = −1.38, false discovery rate *q* = 0.04).

To gain insight into the molecular determinants of synaptic transmission and plasticity alterations in OPHN1 adult deficient mice, we then performed transcriptomic analysis from the hippocampi of WT and KO mice. Gene set enrichment analysis (GSEA) comparing the glutamatergic signalization gene expression profile (69 genes) was performed on microarray transcriptomes from the hippocampi of WT and KO mice. Genes involved in the regulation of glutamatergic transmission were more enriched in WT compared to KO, consistent with the reduction in excitatory neurotransmission in KO mice (Figure 4A).

We performed additional analysis by GSEA to explore whether one synaptic compartment was more affected than another. On one hand, the postsynaptic gene set expression profile (252 genes) was used to compared WT and KO littermates, and no statistical difference was detected (Figure 4B). On the other hand, the presynaptic gene set expression profile (465 genes) was preferentially enriched in WT compared to KO (Figure 4C). Lastly, we used a dataset of 140 genes involved in neurotransmission and found that they were significantly enriched in WT mice (Figure 4D). Among these 140 genes, the 17 (Figure 4D, yellow highlight) contributing mostly to the enrichment (Figure 4E) are involved in exocytosis according to Gene Ontology analyses using the Database for Annotation, Visualization, and Integrated Discovery (DAVID). Altogether, these results point to a presynaptic origin of the altered excitatory synaptic transmission, with a downregulation of genes involved in neurosecretion.

## 4. Discussion

In the present study, we showed that OPHN1 deficiency induces, during postnatal development, a progressive alteration in synaptic transmission and plasticity resulting from presynaptic defects. Furthermore, we show that, whereas basal excitatory synaptic transmission and LTP are reduced in KO adult mice, the number of excitatory synapses is increased. The dichotomy between the number of excitatory synapses and synapse efficacy suggests that a proportion of synapses are immature, as previously shown in vitro in cultures of neurons [11]. Alternatively, excitatory neurons may compensate for the lower synaptic efficiency by increasing the number of presynaptic contacts. Interestingly, immature synapses do not release neurotransmitters in response to presynaptic action potentials [24,25], indicating a very low presynaptic release probability. Our data show that the release probability of synapses is decreased in KO mice, further suggesting the presence of immature synapses. OPHN1 interacts with key actors of clathrin-mediated endocytosis, such as amphiphysin I and II, CIN85, and endophilin [26]. As a result, vesicle endocytosis, i.e., vesicle recycling and pool replenishment, has been reported to be altered in the presynaptic compartment of KO mice. This impairment results from changes in the RhoA pathway, as inhibition of this pathway rescues the endocytosis defects in OPHN1-deficient mice [16,26]. In addition, we found a reduced PTP in KO mice. PTP involves presynaptic binding of calcium to calmodulin to trigger the release of synaptic vesicles (SV), particularly the readily releasable pool (RRP) of vesicles, which is replenished from a larger reserve pool of vesicles. The RRP is thought to represent the vesicles docked at the nerve terminal membrane in active zones. The size of the RRP is a key determinant of synaptic efficacy, and fluctuations in pool size determine in part the strength of synapses during stimulation [27]. In accordance to our results, the RRP is reduced in the hippocampus of OPHN1-deficient mice [17]. On the other hand, the GSEA analysis revealed that presynaptic genes involved in neurotransmitter release are downregulated in OPHN1-deficient mice. This latter signature may be related to the previously described OPHN1 function in exocytotic fusion in the neuroendocrine cells [28].

Lastly, the identification of different alterations in neurotransmission according to postnatal stages is very promising since it provides some therapeutic opportunity to treat patients between 1 and 4 years, corresponding to 1 and 3 months studied here in mouse (https://www.translatingtime.org/, accessed on 10 April 2013). It is noteworthy that, previous preclinical trials using fasudil, a ROCK and PKA inhibitor, failed to restore the deficit in spatial memory in Ophn1^−/y^ mice older than 4 months, suggesting that the defects in excitatory synaptic transmission in the hippocampus may not be reversed by modulating the Rho GTPase pathway [18]. It would be interesting to repeat these assays at early stages between 1 and 3 months or to use molecules to temporally restore brain plasticity, such as fluoxetine [29] in older animals, together with fasudil to treat the unbalanced RhoGTPase pathway while stimulating the animal during the trial acquisition.

## Data Availability

Transcriptional raw and normalized data are available at https://www.ncbi.nlm.nih.gov/geo/query/acc.cgi?acc=GSE190891 (accessed on 24 December 2021). The data that support the findings of this study are available from the corresponding author upon reasonable request.

## References

[1] Milani D., Ronzoni L., Esposito S. (2015). Genetic Advances in Intellectual Disability. J. Pediatr. Genet..

[2] Salvador-Carulla L., Reed G.M., Vaez-Azizi L.M., Cooper S.-A., Martinez-Leal R., Bertelli M., Adnams C., Cooray S., Deb S., Akoury-Dirani L. (2011). Intellectual developmental disorders: Towards a new name, definition and framework for “mental retardation/intellectual disability” in ICD-11. World Psychiatry.

[3] Ropers H.-H., Hamel B.C.J. (2005). X-linked mental retardation. Nat. Rev. Genet..

[4] Humeau Y., Gambino F., Chelly J., Vitale N. (2009). X-linked mental retardation: Focus on synaptic function and plasticity. J. Neurochem..

[5] Pavlowsky A., Chelly J., Billuart P. (2012). Emerging major synaptic signaling pathways involved in intellectual disability. Mol. Psychiatry.

[6] Valnegri P., Sala C., Passafaro M. (2012). Synaptic dysfunction and intellectual disability. Adv. Exp. Med. Biol..

[7] Billuart P., Bienvenu T., Ronce N., des Portes V., Vinet M.C., Zemni R., Crollius H.R., Carrié A., Fauchereau F., Cherry M. (1998). Oligophrenin-1 encodes a rhoGAP protein involved in X-linked mental retardation. Nature.

[8] Piton A., Gauthier J., Hamdan F.F., Lafrenière R.G., Yang Y., Henrion E., Laurent S., Noreau A., Thibodeau P., Karemera L. (2011). Systematic resequencing of X-chromosome synaptic genes in autism spectrum disorder and schizophrenia. Mol. Psychiatry.

[9] Zanni G., Saillour Y., Nagara M., Billuart P., Castelnau L., Moraine C., Faivre L., Bertini E., Durr A., Guichet A. (2005). Oligophrenin 1 mutations frequently cause X-linked mental retardation with cerebellar hypoplasia. Neurology.

[10] Guo D., Yang X., Shi L. (2020). Rho GTPase Regulators and Effectors in Autism Spectrum Disorders: Animal Models and Insights for Therapeutics. Cells.

[11] Khelfaoui M., Denis C., van Galen E., de Bock F., Schmitt A., Houbron C., Morice E., Giros B., Ramakers G., Fagni L. (2007). Loss of X-Linked Mental Retardation Gene Oligophrenin1 in Mice Impairs Spatial Memory and Leads to Ventricular Enlargement and Dendritic Spine Immaturity. J. Neurosci..

[12] Allegra M., Spalletti C., Vignoli B., Azzimondi S., Busti I., Billuart P., Canossa M., Caleo M. (2017). Pharmacological rescue of adult hippocampal neurogenesis in a mouse model of X-linked intellectual disability. Neurobiol. Dis..

[13] Nadif Kasri N., Nakano-Kobayashi A., Malinow R., Li B., Van Aelst L. (2009). The Rho-linked mental retardation protein oligophrenin-1 controls synapse maturation and plasticity by stabilizing AMPA receptors. Genes Dev..

[14] Nakano-Kobayashi A., Tai Y., Nadif Kasri N., Van Aelst L. (2014). The X-linked Mental Retardation Protein OPHN1 Interacts with Homer1b/c to Control Spine Endocytic Zone Positioning and Expression of Synaptic Potentiation. J. Neurosci..

[15] Redolfi N., Galla L., Maset A., Murru L., Savoia E., Zamparo I., Gritti A., Billuart P., Passafaro M., Lodovichi C. (2016). Oligophrenin-1 regulates number, morphology and synaptic properties of adult-born inhibitory interneurons in the olfactory bulb. Hum. Mol. Genet..

[16] Khelfaoui M., Pavlowsky A., Powell A.D., Valnegri P., Cheong K.W., Blandin Y., Passafaro M., Jefferys J.G.R., Chelly J., Billuart P. (2009). Inhibition of RhoA pathway rescues the endocytosis defects in Oligophrenin1 mouse model of mental retardation. Hum. Mol. Genet..

[17] Powell A.D., Gill K.K., Saintot P.-P., Jiruska P., Chelly J., Billuart P., Jefferys J.G.R. (2012). Rapid reversal of impaired inhibitory and excitatory transmission but not spine dysgenesis in a mouse model of mental retardation. J. Physiol..

[18] Meziane H., Khelfaoui M., Morello N., Hiba B., Calcagno E., Reibel-Foisset S., Selloum M., Chelly J., Humeau Y., Riet F. (2016). Fasudil treatment in adult reverses behavioural changes and brain ventricular enlargement in Oligophrenin-1 mouse model of intellectual disability. Hum. Mol. Genet..

[19] Chever O., Dossi E., Pannasch U., Derangeon M., Rouach N. (2016). Astroglial networks promote neuronal coordination. Sci. Signal..

[20] Renaud J., Dumont F., Khelfaoui M., Foisset S.R., Letourneur F., Bienvenu T., Khwaja O., Dorseuil O., Billuart P. (2015). Identification of intellectual disability genes showing circadian clock-dependent expression in the mouse hippocampus. Neuroscience.

[21] Valnegri P., Khelfaoui M., Dorseuil O., Bassani S., Lagneaux C., Gianfelice A., Benfante R., Chelly J., Billuart P., Sala C. (2011). A circadian clock in hippocampus is regulated by interaction between oligophrenin-1 and Rev-erbα. Nat. Neurosci..

[22] Subramanian A., Tamayo P., Mootha V.K., Mukherjee S., Ebert B.L., Gillette M.A., Paulovich A., Pomeroy S.L., Golub T.R., Lander E.S. (2005). Gene set enrichment analysis: A knowledge-based approach for interpreting genome-wide expression profiles. Proc. Natl. Acad. Sci. USA.

[23] Busti I., Allegra M., Spalletti C., Panzi C., Restani L., Billuart P., Caleo M. (2020). ROCK/PKA inhibition rescues hippocampal hyperexcitability and GABAergic neuron alterations in Oligophrenin-1 Knock-out mouse model of X-linked intellectual disability. J. Neurosci..

[24] Crawford D.C., Mennerick S. (2012). Presynaptically silent synapses: Dormancy and awakening of presynaptic vesicle release. Neuroscientist.

[25] Hanse E., Seth H., Riebe I. (2013). AMPA-silent synapses in brain development and pathology. Nat. Rev. Neurosci..

[26] Nakano-Kobayashi A., Kasri N.N., Newey S.E., Van Aelst L. (2009). The Rho-linked mental retardation protein OPHN1 controls synaptic vesicle endocytosis via endophilin A1. Curr. Biol..

[27] Balakrishnan V., Srinivasan G., von Gersdorff H. (2010). Post-tetanic potentiation involves the presynaptic binding of calcium to calmodulin. J. Gen. Physiol..

[28] Houy S., Estay-Ahumada C., Croisé P., Calco V., Haeberlé A.-M., Bailly Y., Billuart P., Vitale N., Bader M.-F., Ory S. (2015). Oligophrenin-1 Connects Exocytotic Fusion to Compensatory Endocytosis in Neuroendocrine Cells. J. Neurosci..

[29] Ohira K., Hagihara H., Miwa M., Nakamura K., Miyakawa T. (2019). Fluoxetine-induced dematuration of hippocampal neurons and adult cortical neurogenesis in the common marmoset. Mol. Brain.

